# A rare tumour in the cerebellopontine angle: endolymphatic sac tumour

**DOI:** 10.11604/pamj.2018.31.127.3962

**Published:** 2018-10-19

**Authors:** Abderrahim Elktaibi, Amal Damiri, Issam Rharrassi, Mohamed Reda Elochi, Mohamed Oukabli, Ali Akhaddar, Mohamed Boucetta, Abderrahmanne Al Bouzidi

**Affiliations:** 1 Department of Pathological Anatomy, Mohammed V Military Teaching Hospital, Rabat, Morocco; 2 Department of Neurosurgery, Mohammed V Military Teaching Hospital, Rabat, Morocco

**Keywords:** Endolymphatic sac, papillary tumor, cerebellopontine angle, surgery, radiotherapy

## Abstract

We present a case of a papillary tumour at the cerebellopontine angle in a 54-year-old man. He presented with right-sided ear pain associated with dizziness and hearing loss. The radiological diagnosis was in favor of acoustic neurinoma. Surgical excision was performed and the diagnosis of the endolymphatic sac tumour was made. Endolymphatic tumour is a low grade adenocarcinoma that originates from the endolymphatic sac. The definitive diagnosis requires a combination of clinical features, radiological finding and pathological correlation.

## Introduction

Endolymphatic sac tumor (ELST) is a rare neoplasm with benign histopathological appearance and clinically destructive behavior which occurs in the skull base and frequently invades the posterior petrous bone, the mastoid and cerebellopontine angle (CPA). It is synonymous with Heffner tumor, low grade adenocarcinoma of endolymphatic sac origin, and aggressive papillary middle ear tumor according to the recently published World Health Organization tumor classification [1]. It is extremely rare in the general population and has an association with Von Hippel-Lindau (VHL) disease [2]. Although the endolymphatic sac papillary tumor is a benign tumor, its growth pattern is often invasive [3]. We present a case of endolymphatic sac tumour in a 54-year-old man who was clinically and radiologically diagnosed as acoustic schwannoma and review the available literature on its clinicopathological and therapeutic features.

## Patient and observation

**Figure 1:**
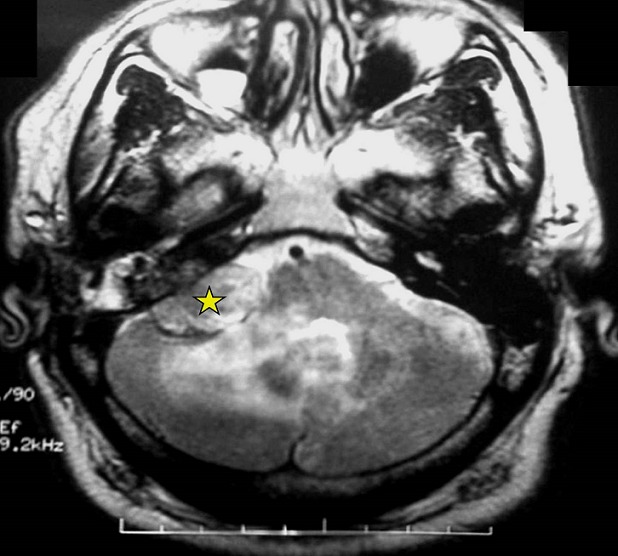
Cerebral axial T2-weighted magnetic resonance imaging showing a well circumscribed multi-lobulated extra-axial mass in the right cerebellopontine angle with cerebellar compression

**Figure 2:**
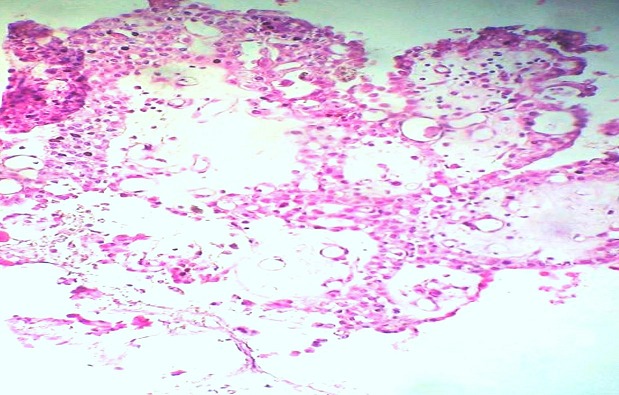
Microscopic appearance of the tumour showing papillary structures lined by a single layer of cuboidal epithelium (Hematoxylin and Eosin × 250)

**Figure 3:**
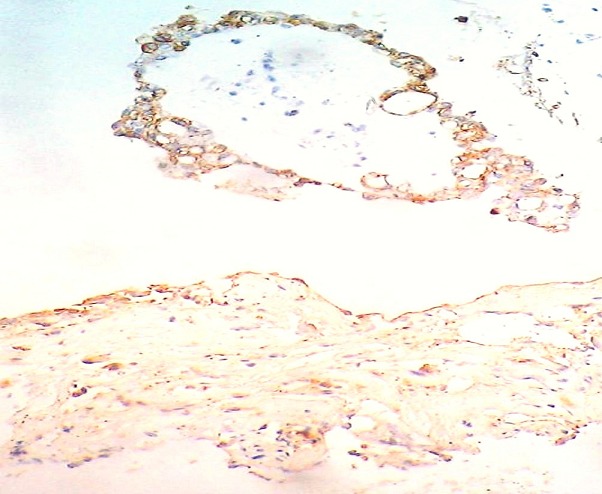
Immunostaining feature: positivity of tumoral cells for epithelial membrane antigen (EMA) (× 250)

A 54-year-old man presented with a chief complaints of progressive hearing loss of approximately 4-year duration. In the two past months, the symptom increased accompanied by headaches, tinnitus and dizziness. Otological examination revealed hearing loss in his right ear associated with incomplete right-sided facial paralysis. There was no history of trauma or surgeries. The left ear was normal. Neither the symptoms nor a family history of VHL disease were found in the patient. On magnetic resonance imaging (MRI), a 3.2×4.1 cm sized multi-lobulated extra-axial mass was detected in the right CPA, compressing the cerebellum. The cranial nerve complexes VII and VIII could not be differentiated from the mass and the radiological diagnosis at that time was acoustic neurinoma (Figure 1). Microscopic examination of the excised material revealed on hematoxylin and eosin staining papillary projections with oedematous and fibrovascular core. The papillae were lined by a single layer of low columnar and cuboidal epithelium. Areas of glandular structures with similar epithelial linings and eosinophilic secretion are noted. The neoplastic cells showed monomorphic nuclei with no mitotic figures, atypia or necrosis seen (Figure 2). Immunohistochemical study was performed and the tumour showed positivity towards cytokeratin, epithelial membrane antigen (EMA), vimentin and focal positivity towards glial fibrillary acidic protein (GFAP) (Figure 3). Stains for thyroid transcription factor-1 (TTF-1), prostate specific antigen, neuron-specific enolase (NSE), chromogranin and synaptophysin were negative. These findings support diagnosis of an endolymphatic sac papillary tumor. The tumor excision was undergone with a sub-occipital CPA approach. No neurologic deficits were observed after surgery. Postoperatively, there was no locoregional recurrence seen 12 months after the operation.

## Discussion

Neoplasm arising from the endolymphatic sac was first described by Hassard [1]. Subsequently, the term low grade adenocarcinoma of endolymphatic sac origin was coined by Heffner in 1989 who found similarities in the anatomical location and histological features of the normal endolymphatic sac to this tumour [4]. A number of incorrect definitions such as primary middle ear adenoma, adenomatous tumors of the middle ear, adeno-cysto-carcinoma, choroidal plexus papilloma, have been applied subsequently to ELST [3]. ELST is reported to be more common in females with a mean presenting age of 45 years. Sensorineural hearing loss, tinnitus and vertigo are the typical presenting symptoms in patients with ELST as seen in our patient. Cranial nerve paralysis including facial nerve palsy and cerebellar disorders develops as the tumor extends into the jugular foramen and cerebellopontine angle [5]. This neoplasm behaves as a slow growing tumor which is locally invasive and exhibits bone destruction. Earlier reported cases of ELST showed no metastasis but recently ELST metastasis to the spine has been observed [6].

The majority of ELST are sporadic while around 15% of patients have an autosomal dominant inherited disorder, von Hippel-Lindau disease. Patients with VHL disease have a germline mutation in the VHL tumor suppressor gene that is responsible for their genetic susceptibility to various neoplasms. Apart from ELST, VHL disease is associated with haemangioblastoma of the central nervous system, choroid plexus papilloma, renal cell carcinoma, phaeochromocytoma and papillary cystadenoma of the epidydimis [2]. Our patient did not have any other associated abnormality on thorough examination. The differential diagnosis of ELST includes other destructive lesions of the temporal bone such as paraganglioma, meningioma, hemangiopericytoma, and metastases. Radiologically, hypervascular mass near the temporal bone is strongly suggestive of a paraganglioma. Typical Zellballen pattern along with immunopositive for chromogranin and synaptophysin distinguishes it from ELST. Rare cases of papillary meningioma have been reported in the temporal bone but they are cytologically anaplastic with areas of necrosis, pleomorphism, and high mitotic activity. Metastatic lesions to the temporal bone may cause difficulty in diagnosis but proper work up along with immunohistochemical stains will help to distinguish between the two [7]. The imaging studies reveal a destructive lesion of the petrous bone which is heterogeneous, space-occupying lesion arising from and invading the temporal bone and even extending into the posterior fossa. Other differential image diagnoses for ELST include acoustic neuroma, which could lead to the destruction of the petrous apex of the temporal bone [8] and compressing the cerebellum.

The present case showed a similar appearance in magnetic resonance imaging. Concerning treatment of this tumor, radical surgical local excision remains the mainstay of current therapy. The best treatment choice is the total removal of the lesion, which may sometimes necessitate sacrifice of cranial nerves, because total resection of the advanced tumors may be impossible due to the anatomic complexity [9], so postoperative radiotherapy was suggested as adjuvant therapy in most cases. Owing to their locally aggressive nature and difficult to extirpate surgically, local radiotherapy should be applied in time, depending on surgical excision status. In addition, radiotherapy may also be suitable as a salvage treatment in recurrent endolymphatic sac tumors [10].

## Conclusion

ELST should be taken into consideration for differential diagnosis of CPA tumors. Detailed clinical and radiographic evaluation is required to direct an appropriate management in every case. Radical excision is feasible using appropriate surgical approach. Early diagnosis, surgical excision and long-term regular follow-up may constitute an efficacious management.

## Competing interests

The authors declare no competing interests.
